# Biomechanical signals and the C-type natriuretic peptide counteract catabolic activities induced by IL-1β in chondrocyte/agarose constructs

**DOI:** 10.1186/ar3459

**Published:** 2011-09-13

**Authors:** Manoj Ramachandran, Prim Achan, Donald M Salter, Dan L Bader, Tina T Chowdhury

**Affiliations:** 1 School of Engineering and Materials Science, Queen Mary University of London, Mile End Road, London E1 4NS, UK; 2 Department of Trauma and Orthopaedics, Barts and The London School of Medicine and Dentistry, Queen Mary University of London, Whitechapel Road, London E1 1BB, UK; 3 Molecular Medicine Centre, Western General Hospital, University of Edinburgh, Crew Road, Edinburgh EH4 2XU, UK

## Abstract

**Introduction:**

The present study examined the effect of C-type natriuretic peptide (CNP) on the anabolic and catabolic activities in chondrocyte/agarose constructs subjected to dynamic compression.

**Methods:**

Constructs were cultured under free-swelling conditions or subjected to dynamic compression with low (0.1 to 100 p*M*) or high concentrations (1 to 1,000 n*M*) of CNP, interleukin-1β (IL-1β), and/or KT-5823 (inhibits cyclic GMP-dependent protein kinase II (PKGII)). Anabolic and catabolic activities were assessed as follows: nitric oxide (NO) and prostaglandin E_2 _(PGE_2_) release, and [^3^H]-thymidine and ^35^SO_4 _incorporation were quantified by using biochemical assays. Gene expression of inducible nitric oxide synthase (iNOS), cyclooxygenase-2 (COX-2), aggrecan, and collagen type II were assessed with real-time quantitative PCR (qPCR). Two-way ANOVA and the *post hoc *Bonferroni-corrected *t *tests were used to examine data.

**Results:**

CNP reduced NO and PGE_2 _release and partially restored [^3^H]-thymidine and ^35^SO_4 _incorporation in constructs cultured with IL-1β. The response was dependent on the concentration of CNP, such that 100 p*M *increased [^3^H]-thymidine incorporation (*P *< 0.001). This is in contrast to ^35^SO_4 _incorporation, which was enhanced with 100 or 1000 n*M *CNP in the presence and absence of IL-1β (*P *< 0.001). Stimulation by both dynamic compression and CNP and/or the PKGII inhibitor further reduced NO and PGE_2 _release and restored [^3^H]-thymidine and ^35^SO_4 _incorporation. In the presence and absence of IL-1β, the magnitude of stimulation for [^3^H]-thymidine and ^35^SO_4 _incorporation by dynamic compression was dependent on the concentration of CNP and the response was inhibited with the PKGII inhibitor. In addition, stimulation by CNP and/or dynamic compression reduced IL-1β-induced iNOS and COX-2 expression and restored aggrecan and collagen type II expression. The catabolic response was not further influenced with the PKGII inhibitor in IL-1β-treated constructs.

**Conclusions:**

Treatment with CNP and dynamic compression increased anabolic activities and blocked catabolic effects induced by IL-1β. The anabolic response was PKGII mediated and raises important questions about the molecular mechanisms of CNP with mechanical signals in cartilage. Therapeutic agents like CNP could be administered in conjunction with controlled exercise therapy to slow the OA disease progression and to repair damaged cartilage. The findings from this research provide the potential for developing novel agents to slow the pathophysiologic mechanisms and to treat OA in the young and old.

## Introduction

In healthy cartilage, chondrocytes mediate matrix remodelling through a balance in the synthesis and degradation of the extracellular matrix components. This constant process is regulated by transient autocrine and paracrine factors, which act through common pathways, involving cytokines, signaling molecules, kinases, and transcription factors, each of which is additionally influenced by mechanical signals [1-6]. However, aging or injury to the joint may trigger mechanical overload and influence these pathways, leading to matrix damage and osteoarthritis (OA) [7,8]. The identification of the signals that are activated during the different stages of the disease process is highly challenging and involves examination of both molecular and mechanical factors. To date, no successful chondroprotective or disease-modifying therapies are available to intervene in this pathological cycle and help to restore joint function. Thus, agents for promoting biophysical and therapeutic strategies to slow the pathophysiologic mechanisms and treat OA are under active investigation.

As an example, the C-type natriuretic peptide (CNP) has recently emerged as an important anabolic regulator of cartilage [9-11]. Indeed, stimulation of chondrocytes with CNP has been reported to increase collagen and proteoglycan synthesis and to enhance cell proliferation [12-14]. Moreover, the guanylyl cyclase B and intracellular 3,5'-cyclic guanosine monophosphate (GC-B/cGMP) pathway was shown to mediate the increase of cell proliferation in rat chondrocytes treated with CNP [15,16]. Upregulation of the GC-B/cGMP system by CNP is essential for cartilage development and involves cyclic GMP-dependent protein kinase II (PKGII) mechanisms in late proliferative and pre-hypertrophic zones of growth-plate cartilage [9,17-19]. Indeed, targeted disruption of the genes encoding CNP and PKGII results in impaired growth of endochondral bones and leads to severe dwarfism and skeletal defects [9,17,18]. Conversely, overexpression of CNP results in skeletal overgrowth and rescued dwarfism in a murine model of human achondroplasia [20]. Consequently, growing evidence suggests that stimulation of CNP signaling may contribute to anabolic events and potentially provide a new therapeutic application for conditions with loss of cartilage matrix and in the treatment of skeletal growth disorders.

Numerous studies have shown that chondrocytes from many species produce nitric oxide (NO) and prostaglandin E_2 _(PGE_2_) release, via induction of the inducible nitric oxide synthase (iNOS) and cyclooxygenase (COX-2) enzymes, in response to interleukin-1β (IL-1β) and tumor necrosis factor alpha (TNF-α) [21-24]. These pro-inflammatory cytokines are involved in the pathogenesis of OA, but their regulation by mechanical signals is necessarily complex. For example, *in vitro *mechanical conditioning experiments demonstrate that mechanical signals, representing a controlled physiological activity, inhibit IL-1β-induced iNOS and COX-2 expression and restore matrix synthesis [25,26]. The opposite effect was found for mechanical signals, which could be interpreted as an excessive or injurious response, and enhanced the IL-1β-induced catabolic response [27]. These findings indicate that mechanical signals that mimic the physiological loading environment of cartilage act in an anti-inflammatory manner and could therefore provide a physical strategy to repair damaged tissue [28,29]. Our data concur with clinical findings that demonstrate the beneficial effects of prescribed rehabilitative therapies for reducing inflammation and improving joint function in patients with knee OA [30-32]. However, no agreement has been achieved on whether controlled exercise therapy could be efficacious in the aging population [33]. Consequently, restoration of chondrocyte function with CNP in combination with physical therapies may promote cartilage health in the OA joint.

These observations raise the possibility for the potential therapeutic effects of both CNP and mechanical stimuli in reducing the cytokine-induced catabolic events in OA. However, very little is known about the molecular mechanisms activated by CNP and their role in stimulating matrix production in OA chondrocytes. No studies have investigated the interactions of mechanical loading with the CNP pathway or whether they compete with catabolic pathways induced by cytokines. The present study therefore examines the effect of CNP and mechanical loading on anabolic and catabolic activities in chondrocyte/agarose constructs stimulated with IL-1β.

## Materials and methods

### Chondrocyte isolation and culture in agarose constructs

Human cartilage was obtained from nine patients (age 35 to 58 years), with ethical approval (East London and The City Research Ethics Committee) and informed patient consent, undergoing total knee arthroplasty at the Royal London Hospital, Barts, and the London NHS Trust, London, UK. Cartilage was removed from the femoral condyles and tibial plateaus. The morphology of the cartilage specimens was graded for gross degenerative changes according to the ICRS classification, and tissues that represent normal (grade 0 or 1) and early (grade 2) OA were used for experiments. Each experimental condition was repeated with chondrocytes from three to four different donors. Cartilage tissue was diced and incubated on rollers for 1 hour at 37°C in Dulbecco Modified Eagle Medium (DMEM) supplemented with 10% (vol/vol) Fetal Calf Serum (FCS) + 2 μ*M *L-glutamine, 5 μg/ml penicillin, 5 μg/ml streptomycin, 20 m*M *Hepes buffer, and 0.05 mg/ml L-ascorbic acid + 700 unit/ml pronase and incubated for a further 16 hours at 37°C in DMEM + 10% FCS, supplemented with 100 units/ml collagenase type XI (Sigma-Aldrich, Poole, UK). The cell suspension was washed and viable chondrocytes counted using a hemocytometer and trypan blue. Cells were finally resuspended in medium at a cell concentration of 8 × 10^6 ^cells/ml by using well-established methods [34,35]. In brief, the cell suspension was added to an equal volume of molten 6% (wt/vol) agarose type VII in Earle Balanced Salt Solutions (EBSS) to yield a final cell concentration of 4 × 10^6 ^cells/ml in 3% (wt/vol) agarose (Sigma-Aldrich, Poole, UK). The chondrocyte/agarose suspension was transferred into a sterile stainless steel mold, containing holes 5 mm in diameter and 5 mm in height and allowed to gel at 4°C for 20 minutes. Constructs were cultured in a defined culture medium comprising DMEM, 0.1 μ*M *dexamethasone, 0.17 m*M *ascorbate, 1 m*M *sodium pyruvate, 0.35 m*M *proline, 5 μg/ml penicillin, 5 μg/ml streptomycin, 20 m*M *Hepes buffer, 2 μ*M *L-glutamine, ITS, and supplements (6.25 μg/ml insulin, 6.25 μg/ml transferrin, 6.25 μg/ml seleneous acid, 5.33 μg/ml linoleic acid, and 1.25 μg/ml bovine serum albumin) at 37°C in 5% CO_2 _for 24 hours (all from Cambrex Bioscience, Wokingham, UK).

### Dose-response effect of CNP in chondrocyte/agarose constructs

The dose-response effect of CNP was examined in constructs cultured under free-swelling conditions to determine the appropriate concentration for mechanical loading studies. Constructs were cultured in 1 ml of defined media supplemented with either low (0, 0.1, 1, 10, 100 p*M*) or high (0, 1, 10, 100, 1000 n*M*) concentrations of CNP in the presence and absence of 10 ng/ml IL-1β and/or 5 μ*M *KT5823 for 48 hours (all from Sigma-Aldrich). KT5823 inhibits PKGII by competing directly with ATP at the catalytic domain. In each case, the medium was additionally supplemented with 1 μCi/ml [^3^H]-thymidine and 10 μCi/ml ^35 ^SO_4 _(both Amersham Biosciences Ltd, Bucks, UK) for the assessment of cell proliferation and proteoglycan synthesis, respectively. At the end of the culture period, the constructs and corresponding media were immediately stored at -20°C before biochemical analysis.

### Application of dynamic compression

In separate experiments, a fully characterized bioreactor compression system (Zwick Testing Machines, Leominster, UK) was used to determine the effect of CNP and dynamic loading on cell metabolism and gene expression in chondroycte/agarose constructs. The bioreactor has been extensively described previously [34-36]. To review briefly, equilibrated constructs were transferred into individual wells of a 24-well culture plate (Costar, High Wycombe, UK) and mounted within the bioreactor. One milliliter of defined media supplemented with 0 or 10 ng/ml IL-1β in the presence and absence of low (100 p*M*) or high (100 n*M*) concentrations of CNP and/or 5 μ*M *KT5823 were introduced into each well. Strained constructs were subjected to dynamic compression ranging from 0 to 15% strain in a sinusoidal waveform at a frequency of 1 Hz. The compression regimen was applied in an intermittent manner, with a profile of 1.5 hour compression followed by a 4.5 hour unstrained period for both the 6 and 48 hour culture periods. This resulted in duty cycles equivalent to 5400 and 43200, respectively. Control constructs were maintained in an unstrained state within the bioreactor system and cultured for the same time period. At the end of the culture period, the constructs and corresponding media were immediately stored at -70°C before analysis.

### RNA isolation, cDNA synthesis, and real-time qPCR

RNA was isolated from chondrocytes cultured in agarose by using protocols described in the QIAquick Spin gel extraction and RNeasy kits, as previously described (Qiagen, West Sussex, UK) [28,37]. By following manufacturer's instructions, Ambion's DNA-*free *DNase treatment and removal reagents were used to eliminate any contaminating DNA from the RNA sample (Ambion Applied Biosystems, Warrington, UK). RNA was quantified on the Nanodrop ND-1000 spectrophotometer (LabTech, East Sussex, UK), and reverse transcription was performed by using manufacturer's protocols from the Enhanced Avian RT First Strand cDNA synthesis kit, oligo(dT)_23 _primer, and a total of 200 ng of RNA (Sigma Genosys, Cambridge, UK). Real-time quantitative PCR assays coupled with LNA probes were performed in 25-μl reaction mixtures containing 1 μl cDNA, 12.5 μl JumpStart *Taq *PCR Master Mix, primer pairs, and probes detailed in Table 1 and nuclease-free PCR-grade water to 25 μl (Sigma Genosys, Cambridge, UK). Each sample was run in duplicate on the 96-well thermal system of the Mx3000P quantitative PCR instrument (Stratagene, Amsterdam, The Netherlands). Thermocycling conditions comprised an initial polymerase activation step at 95°C for 3 minutes, followed by denaturation of 35 cycles at 95°C for 30 seconds, annealing at 55°C for 1 minute, and extension at 72°C for 1 minute. PCR efficiencies for optimal primer pair and probe concentrations were derived from standard curves (*n *= 3) by preparing a 10-fold serial dilution of cDNA from a sample that represented the untreated control at time-zero conditions. The real-time PCR efficiencies (E) of amplification for each target was defined according to the relation, E = 10^[-1/slope]^. The *R^2 ^*value of the standard curve exceeded 0.9998 and revealed efficiency values presented in Table 1.

**Table 1:** Description of the LNA probe and primer sequences used to quantify gene expression

Gene	Gene ID	Sequences	Product length	Efficiency
iNOS	4843	*Probe: *5'-FAM-ACT**T**CTTTC**C**CG**T**CTCC-BHQ1-3'	305	1.99 ± 0.8
		*Sense: *5'-TCCAGATAAGTGACATAAGTG-3'		
		*Antisense: *5'-CAGCTTGACCAGAGATTC-3'		
COX-2	5743	*Probe: *5'-AAA**C**TG**C**TC**A**AC**A**CCG-BHQ1-3'	216	1.99 ± 2.8
		*Sense: *5'- GGACAGGATTCTATGGAG-3'		
		*Antisense: *5'- GGATGTCAACACATAACTC-3'		
Aggrecan	176	*Probe: *5-'FAM-CCA**A**CT**C**TT**C**AA**G**GTGA-BHQ1-3'	109	1.98 ± 0.4
		*Sense: *5'-GACTGAAGTTCTTGGAGAA-3'		
		*Antisense: *5'-CACGAAAACCCAGAGTAA-3'		
Collagen type II	1280	*Probe: *5'-FAM-TCT**G**TC**T**CC**T**TG**C**TTGCCA-BHQ1-3'	200	1.99 ± 0.9
		*Sense: *5'-GGAGTCAAGGGTGATCGT-3'		
		*Antisense: *5'-CTTGTGCACCAGCTTCTC-3'		
GAPDH	2597	*Probe: *5'-HEX-CAG**T**CA**G**CC**G**CA**T**CTTCT-BHQ1-3'	160	1.98 ± 4.3
		*Sense: *5'-TCTCTGCTCCTCCTGTTC-3'		
		*Antisense: *5'-CGCCCAATACGACCAAAT-3'		

Primers used in PCR experiments with Locked Nucleic Acid (LNA) probes produced amplicons between 109 and 305 base pairs and efficiency values between 1.98 and 1.99. Probes contain fluorescein (FAM) or 6-carboxyhexafluorescein (HEX) as the 5'-reporter dye and Black Hole Quencher 1 (BHQ1) as the 3'-quencher. Nucleotides highlighted in bold denote the LNA base.

Fluorescence data were collected during the annealing stage of amplification, and data were analyzed on the MxPro qPCR software (version 3, Stratagene). Baselines and thresholds were automatically set by the RG-3000 qPCR software and used after manual inspection. The cycle threshold (C_t_) value for each duplicate reaction was expressed as the mean value, and the results were exported into Microsoft Excel for further analysis. The data obtained by PCR assay for GAPDH were validated as a reference gene by displaying the C_t _values as box-and-whisker plots, and the distribution examined under mechanical loading conditions (data not shown). The C_t _values for GAPDH remained stable, with no changes detected under all culture conditions, suggesting its suitability as a reference gene. Relative quantification of iNOS, COX-2, aggrecan, and collagen type II signals were accomplished by normalizing each target to the reference gene, GAPDH, and to the calibrator sample by a comparative C_t _approach. For each sample, the ratio of target ΔCt and reference ΔCt was calculated, as previously described [28,37].

### Biochemical analysis

The production of NO was determined in media by converting nitrate to nitrite by using 1 unit/ml nitrate reductase in 40 μ*M *NAPDH, 500 μ*M *glucose 6-phosphate, 160 unit/ml glucose 6-phosphate dehydrogenase and 20 m*M *Tris-HCL for 15 minutes at 37°C. Total nitrite was assayed spectrophotometrically at 540 nm by using the Griess reaction. PGE_2 _production was measured in the culture media by using a high-sensitivity enzyme immunoassay according to manufacturer's instructions (Amersham Biosciences Ltd, Bucks, UK). [^3^H]-thymidine incorporation was measured in constructs digested overnight at 37°C with 10 U/ml agarase followed by 1 hour at 60°C with 2.8 U/ml papain (both Sigma Chemical Co., Poole, UK) and analyzed with 10% trichloroacetic acid precipitation onto filters by using a Millipore Multiscreen system (Millipore, Watford, UK), as previously described [29,34,35]. Incorporation of ^35^SO_4 _was determined in both agarase/papain digests and the culture media by using the Alcian blue precipitation method, as previously described [29,34,35]. Total DNA content remained stable throughout the culture conditions was assayed by using the Hoescht dye 33258 in agarose/papain digests.

### Statistics

For dose-response studies, data represent the mean and SEM values of six replicates from three separate experiments. For the mechanical loading experiments, biochemical and gene-expression data represent the mean and SEM values of eight replicates from three separate experiments. Statistical analysis was performed with a two-way analysis of variance (ANOVA) and the multiple *post hoc *Bonferroni-corrected *t *tests to compare differences between the various treatment groups, as indicated in the figure legend. For gene-expression data, ratio values were log transformed before analysis by a two-way ANOVA and the *post hoc *Bonferroni-corrected *t *test. In all cases, a level of 5% was considered statistically significant (*P *< 0.05).

## Results

### CNP differentially regulates cell metabolism in a dose-dependent manner

**Figure 1:**
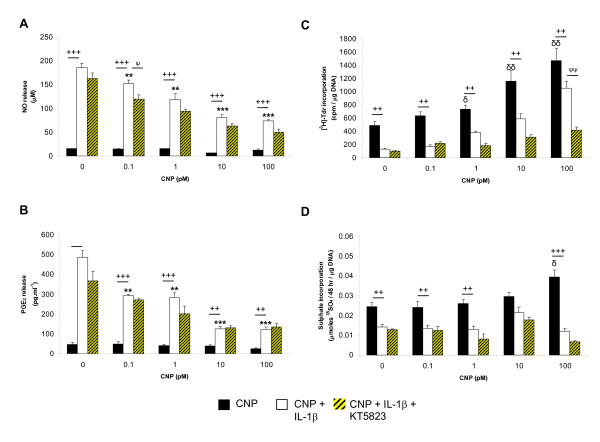
**Dose-response effect of low concentrations of CNP**. Chondrocyte/agarose constructs were cultured under free-swelling conditions with 0.1 to 100 p*M *CNP and 0 or 10 ng/nl IL-1β and/or 5 μ*M *KT5823 for 48 hours. **(a) **NO release; **(b) **prostaglandin E_2 _(PGE_2_) release; **(c) **[^3^H]-thymidine; and **(d) **^35^SO_4 _incorporation (*n *= 6). (δ), significant comparisons for untreated versus CNP; (*), significant comparisons for IL-1β versus IL-1β + CNP; (+), significant comparisons for untreated versus IL-1β + CNP; (ψ), significant comparisons for IL-1β versus IL-1β + CNP + KT5823.

Chondrocytes cultured in agarose constructs produce significant amounts of NO and PGE_2 _release in response to IL-1β (both *P *values < 0.001; Figure 1a and 1b, respectively). To examine whether CNP and the selective PKGII inhibitor could influence the IL-1β-induced NO and PGE_2 _release, constructs were cultured with IL-1β and CNP at concentrations ranging from 0.1 to 100 p*M *in the presence and absence of KT5823. It was evident that CNP reduced IL-1β induced NO and PGE_2 _release in a dose-dependent manner, with maximal inhibition at 10 and 100 p*M *when compared with IL-1β-treated constructs (all *P *< 0.001). Treatment with IL-1β and the PKGII inhibitor did not influence NO and PGE_2 _levels in constructs cultured with CNP (Figures 1a and 1b). In the absence of IL-1β, CNP increased [^3^H]-thymidine incorporation in a dose-dependent manner (*P *< 0.01; Figure 1c), whereas this effect of CNP was reduced by IL-1β and/or KT5823 (*P *< 0.01). At 100 p*M*, CNP increased ^35^SO_4 _incorporation (*P *< 0.05; Figure 1d), and this effect was reduced by IL-1β (*P *< 0.001) and not further influenced with KT5823.

**Figure 2:**
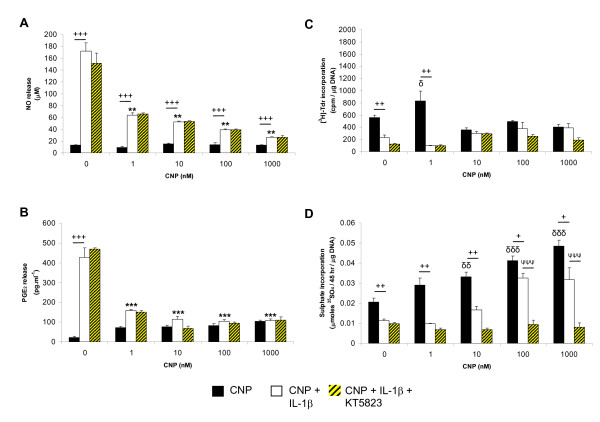
**Dose-response effect of high concentrations of CNP**. Chondrocyte/agarose constructs were cultured under free-swelling conditions with 1 to 1000 n*M *CNP and 0 or 10 ng/nl IL-1β, and/or 5 μ*M *KT5823 for 48 hours. **(a) **NO release; **(b) **prostaglandin E_2 _(PGE_2_) release; **(c) **[^3^H]-thymidine; and **(d) **^35^SO_4 _incorporation (*n *= 6). (δ), significant comparisons for untreated versus CNP; (*), significant comparisons for IL-1β versus IL-1β + CNP; (+), significant comparisons for untreated versus IL-1β + CNP; (ψ), significant comparisons for IL-1β versus IL-1β + CNP + KT5823.

Figure 2 presents the dose-response effects of CNP at concentrations ranging from 0.1 to 1000 n*M *in constructs cultured with IL-1β and/or KT5823 (Figure 2). IL-1β-induced NO and PGE_2 _release was reduced by CNP in a dose-dependent manner (Figure 2a). However, the effect was most pronounced in the presence of 1 n*M *CNP, which completely abolished the IL-1β-induced PGE_2 _release (*P *< 0.001; Figure 2b). Treatment with CNP and the PKGII inhibitor had no further effect on the reduction of IL-1β-induced release of both NO and PGE_2_. At 1 n*M*, CNP increased [^3^H]-thymidine incorporation when compared with untreated controls (*P *< 0.05; Figure 2c). The presence of IL-1β inhibited [^3^H]-thymidine incorporation and the response was not significantly influenced by CNP and/or the PKGII inhibitor (Figure 2c). In contrast, CNP increased ^35^SO_4 _incorporation in a dose-dependent manner with maximal stimulation at 100 and 1000 n*M *(*P *< 0.001; Figure 2d). The presence of IL-1β inhibited ^35^SO_4 _incorporation, and the levels were enhanced with CNP at 100 and 1000 n*M*, only. However, stimulation of ^35^SO_4 _incorporation by CNP in IL-1β-treated constructs was abolished in the presence of the PKGII inhibitor (*P *< 0.001; Figure 2d).

### CNP and dynamic compression counteract IL-1β-induced NO and PGE_2 _release and restore cell proliferation and proteoglycan synthesis

**Figure 3:**
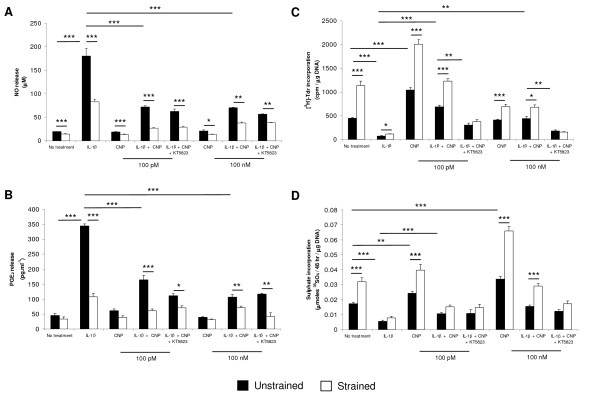
**Effect of CNP and dynamic compression on NO (a) and prostaglandin E_2 _(PGE_2_) release (b), [^3^H]-thymidine (c), and ^35^SO_4 _incorporation (d)**. Chondrocyte/agarose constructs were cultured with 0 or 10 ng/nl IL-1β and either 100 p*M *CNP or 100 n*M *CNP and/or 5 μ*M *KT5823 for 48 hours (*n *= 8). (*), significant comparisons in unstrained and strained constructs for the multiple treatment conditions. All other comparisons (not indicated) were not significant.

In separate experiments, the effects of CNP and dynamic compression were examined in constructs cultured with IL-1β and/or the PKGII inhibitor by using either low (100 p*M*) or high (100 n*M*) concentrations of the peptide (Figure 3). In the absence and presence of CNP, dynamic compression reduced NO release (*P *< 0.001; Figure 3a) but had no significant effect on PGE_2 _levels (Figure 3b). In unstrained constructs, the presence of IL-1β increased NO and PGE_2 _release, and the response was reduced by dynamic compression, CNP, and/or both stimuli (all *P *< 0.001). Stimulation with CNP and/or dynamic compression in the presence of the PKGII inhibitor further reduced NO and PGE_2 _release in IL-1β-treated constructs (both *P *< 0.01; Figure 3a and 3b, respectively). In contrast, dynamic compression increased [^3^H]-thymidine incorporation in the presence and absence of CNP (*P *< 0.001; Figure 3c). This effect was inhibited with IL-1β and could be reversed by stimulation with 100 p*M *CNP, dynamic compression, or both. The presence of the PKGII inhibitor blocked CNP-induced stimulation of [^3^H]-thymidine incorporation in IL-1β-treated constructs. The opposite effect was found for ^35^SO_4 _incorporation, whereby stimulation with 100 n*M *CNP and dynamic compression induced the greatest response when compared with untreated controls or constructs cultured with 100 n*M *CNP (*P *< 0.001; Figure 3d). The IL-1β-induced inhibition of ^35^SO_4 _incorporation was reversed by both 100 n*M *CNP and dynamic compression (*P *< 0.001) and inhibited with KT5823 (Figure 3d).

### CNP and dynamic compression modulate IL-1β-induced gene expression

**Figure 4:**
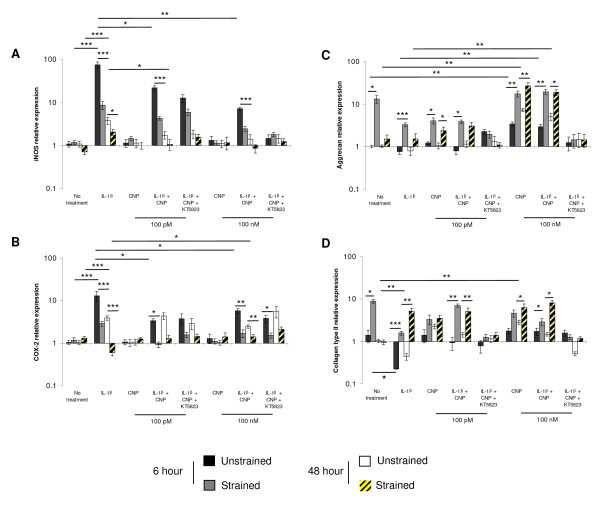
**Effect of CNP and dynamic compression on gene expression**. Chondrocyte/agarose constructs were cultured with 0 or 10 ng/nl IL-1β and either 100 p*M *CNP or 100 n*M *CNP and/or 5 μ*M *KT5823 for 6 and 48 hours (*n *= 8). **(a) **iNOS, **(b) **COX-2, **(c) **aggrecan, and **(d) **collagen type II. (*), significant comparisons in unstrained and strained constructs for the multiple treatment conditions as shown. All other comparisons (not indicated) were not significant.

To investigate the effect of CNP on the expression of catabolic and anabolic genes, constructs were subjected to dynamic compression over 6 and 48 hour period in the presence and absence of low (100 p*M*) and high (100 n*M*) concentrations of the peptide (Figure 4). In unstrained constructs, IL-1β induced iNOS and COX-2 expression at 6 and 48 hours (all *P *< 0.001; Figure 4a and 4b, respectively). At 6 and 48 hours, the IL-1β-induced iNOS and COX-2 expression was inhibited by dynamic compression (both *P *< 0.001) or by the presence of low (both *P *< 0.01) or high concentrations of CNP (both *P *< 0.01). A combination of dynamic compression and CNP reduced iNOS and COX expression at 6 hours, with levels returning to basal values with IL-1β at 48 hours. This effect was not significantly influenced further with KT5823 in constructs cultured with IL-1β and CNP. In contrast, dynamic compression increased aggrecan and collagen type II expression at 6 hours but not at 48 hours (both *P *< 0.05; Figure 4c and 4d, respectively). In unstrained constructs, stimulation with CNP increased aggrecan and collagen type II expression in a concentration-dependent manner and the effect was further enhanced with dynamic compression at either 6 or 48 hours. In unstrained constructs, IL-1β inhibited aggrecan and collagen type II expression and the effect was reversed with dynamic compression (*P *< 0.001), 100 n*M *CNP (*P *< 0.05), or both at 6 (*P *< 0.01) and 48 hours (*P *< 0.05) for aggrecan expression. In contrast, stimulation by 100 p*M *or 100 n*M *CNP and dynamic compression reversed the IL-1β-induced inhibition of collagen type II expression at 6 (both *P *< 0.01) and 48 hours (both *P *< 0.05; Figure 4d). The compression-induced stimulation of aggrecan and collagen type II expression was inhibited with KT5823 in constructs cultured with IL-1β and CNP.

## Discussion

CNP is expressed in the growth plate and regulates endochondral ossification through increased cell proliferation and hypertrophy and stimulates production of cartilage matrix proteins [13,14,19,38,39]. The molecular mechanisms that control these processes are not completely understood. In addition, it is unclear whether the effects of CNP in adult cartilage are influenced by mechanical signals that modulate matrix synthetic activity and inflammatory pathways. However, the response to mechanical signals, in part, is dependent on the type of mechanical loading regime, its duration, and whether loading was applied during the early or late stage of the disease process. Consequently, stimulation of chondrocytes with natriuretic peptides and mechanical signals may potentially serve to modulate cell proliferation and increase matrix synthesis in the OA joint. The present study therefore examined the effect of CNP and mechanical signals in an *in vitro *inflammatory chondrocyte/agarose model.

Initial studies examined the dose-response effect of CNP on cell proliferation and proteoglycan synthesis and whether the peptide could stimulate anabolic activities by blocking the catabolic mediators induced by IL-1β. In the absence of the cytokine, low concentrations of CNP (10 to 100 p*M*) increased cell proliferation without affecting proteoglycan synthesis in chondrocyte/agarose constructs. The opposite effect was found for high concentrations of CNP (100 to 1000 n*M*), which increased proteoglycan synthesis without any significant change in cell proliferation. Our data are in agreement with previous studies that demonstrate differential effects of CNP in chondrocytes cultured in monolayer [13,14]. Other studies have shown that stimulation by CNP increased cell proliferation in growth-plate chondrocytes and chondrogenic cell lines and enhanced GAG synthesis and cell-adhesion molecules in mesenchymal cells [39-41]. In addition, CNP increased the expression of genes involved in proteoglycan synthesis and inhibited the expression of proteinase enzymes involved in matrix breakdown [12,39,41]. CNP therefore plays a significant role in regulating chondrocytes and contributes to the structural properties of cartilage tissue [15,40,42]. However, CNP has never been implicated in OA and the importance of this pathway in chondrocytes is not known. In the present study, biochemical analysis revealed that under IL-1β conditions, CNP inhibits NO and PGE_2 _production in a dose-dependent manner and restores cell proliferation and proteoglycan synthesis. The reparative effect involved PKGII-dependent mechanisms and was influenced by the concentration of CNP, resulting in greater levels of matrix synthesis with nanomolar concentrations in IL-1β-treated constructs. In contrast, picomolar concentrations of CNP increased cell proliferation in the presence of IL-1β, and the response was blocked with the PKGII inhibitor. A recent study showed that CNP can induce hypertrophy in chondrocytes, which may contribute to OA disease progression [43,44]. In the present study, stimulation with CNP resulted in differential effects, such that low doses increased gene expression of type I collagen, type × collagen, and MMP-13 without affecting collagen type II mRNA (Table 2). In contrast, high concentrations had the opposite effect and increased type II collagen gene expression and inhibited type I collagen and MMP-13 in free-swelling constructs (Table 2). Whilst the CNP and PKGII pathways might be used in the treatment of growth retardation, concentration-dependent effects of CNP should be further addressed to ensure that their potential damaging effects of CNP are not evoked in cartilage.

**Table 2:** Effect of CNP on gene expression in chondrocyte/agarose constructs

					100 p*M*						100 n*M*					
	UT		IL-1β		CNP		IL-1β + CNP		IL-1β + CNP + KT5823		CNP		IL-1β + CNP		IL-1β + CNP + KT5823	
Culture period (hr)	6	48	6	48	6	48	6	48	6	48	6	48	6	48	6	48
Collagen type I	^a^	^a^	ND	ND	↑^b^	↑^c^	ND	ND	ND	ND	^a^	↓^d^	ND	ND	ND	ND
Collagen type II	↑^b^	^a^	↓^d^	↓^d^	^a^	^a^	^a^	^a^	^a^	^a^	^a^	↑^c^	↓^d^	↓^d^	^a^	^a^
Col X	^a^	^a^	^a^	^a^	↑^b^	↑^c^	↑ ^b^	↑ ^c^	↓ ^d^	↓^d^	^a^	^a^	^a^	^a^	^a^	^a^
Sox-9	^a^	^a^	ND	ND	^a^	^a^	ND	ND	ND	ND	↑^b^	↑^c^	ND	ND	ND	ND
MMP-13	^a^	^a^	↑^b^	↑^c^	↑^b^	↑^b^	↑^b^	↑^c^	↓^d ^	↓^d^	^a^	^a^	↓^d^	↓^d^	^a^	^a^

^a^No significant change; ↑^b^, a significant increase (*P *< 0.05); ↑^c^, a significant increase (*P *< 0.01); ↓^d^, a significant decrease (*P *< 0.05); ND, not determined. Chondrocyte/agarose constructs were cultured with low (100 p*M*) or high (100 n*M*) concentrations of CNP with 0 or 10 ng/ml IL-1β and/or 5 μ*M *KT5823 for 6 and 48 hours (*n *= 8). Note that statistical comparisons were determined for the following samples: Time = 0 versus 6 or 48 hr (UT); UT versus IL-1β; UT versus CNP; IL-1β versus IL-1β + CNP; IL-1β + CNP versus IL-1β + CNP + KT5823.

Both CNP and NO stimulate the synthesis of cGMP and lead to activation of common downstream pathways, involving PKGII. In the present study, the PKGII inhibitor did not influence the levels of NO and PGE_2 _release following CNP and/or IL-1β treatment, implicating alternative mechanisms. For example, the cGMP pathways are likely to cross-talk with the catabolic pathways because of elevated levels of NO induced by IL-1β. More specifically, NO binds to the heme-containing soluble protein, guanylyl cyclase (sGC), and stimulates cGMP levels, which contribute to the production of PKG and cGMP-regulated phosphodiesterase (PDE) subtypes [45]. NO will change the function of other heme-containing proteins, such as COX-2 and increase PGE_2 _production. In a previous study, overstimulation of the PKGI pathway was shown to block IGF-1-induced proteoglycan synthesis in chondrocyte monolayers [46]. Indeed, it was previously reported with human chondrocytes that enhanced cGMP levels lead to alterations in the PDE5 subtype and matrix breakdown. This effect was mediated by the induction of iNOS and NO release with IL-1β [47]. This is in contrast to chemical inhibitor studies for PDE4, which showed a partial inhibition of NO release in OA chondrocytes [48]. Thus, pharmacologic inhibitors selective for PDE subtypes may reduce the catabolic response in chondrocytes. Some evidence indicates a functional role of PDE4 in downregulating the IGF-1-binding proteins, which are known to contribute to OA pathogenesis [49-51]. The action of elevated cytokine-induced NO/cGMP pathways and complex interplay with CNP is unknown and requires further investigation.

**Figure 5:**
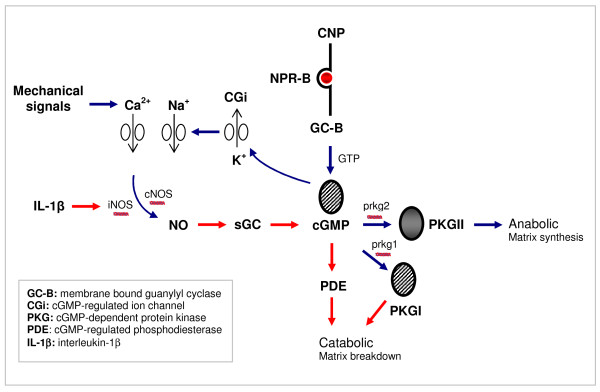
**Proposed signaling interactions between CNP and mechanical signals in chondrocytes**. C-type natriuretic peptide (CNP) binding to the natriuretic peptide receptor-B (NPR-B) activates the extracellular domain of guanylyl cyclase B (GC-B), leading to increased levels of 3,5'-cyclic guanosine monophosphate (cGMP). The accumulation of cGMP levels modulates the downstream activities of cGMP-dependent protein kinases (PKGI and II), cGMP-regulated ion channels (CGi), and cGMP-regulated phosphodiesterase (PDE) subtypes. PKGII mediates matrix synthesis augmented by mechanical signals that influence CGi ion channels. However, the cGMP pathways are likely to crosstalk with the catabolic pathways because of elevated levels of nitric oxide (NO) induced by interleukin-1β (IL-1β). More specifically, NO binds to the heme-containing soluble protein, guanylyl cyclase (sGC), and stimulates cGMP levels, which contribute to the production of PKGI or PDE subtypes, leading to matrix breakdown.

It is well established that NO and PGE_2 _accelerate chondrocyte-mediated matrix degradation, inflammation, and apoptosis [1,52]. NO is also an important signaling molecule in cartilage in response to different stimuli, including pro-inflammatory cytokines and mechanical signals [7,26,28]. We therefore examined whether mechanical signals could influence NO and PGE_2 _levels in chondrocyte/agarose constructs cultured with IL-1β and either low (100 p*M*) or high (100 n*M*) concentrations of CNP. In the presence and absence of IL-1β, stimulation with mechanical signals and CNP induced the expression of aggrecan and collagen type II and increased cell proliferation and proteoglycan synthesis in a concentration-dependent manner. The anabolic response was blocked by the PKGII inhibitor. It is plausible that PKGII represents the principal mediators of cGMP signals and therefore has a positive role in cartilage homeostasis. In the presence of IL-1β, both mechanical signals and CNP reduced iNOS and COX-2 expression and NO and PGE_2 _production. This is the first study to show that CNP and mechanical signals block catabolic activities and rescue anabolic events in chondrocyte/agarose constructs cultured with IL-1β. These findings open the possibility of using CNP in the treatment of damaged cartilage in conjunction with controlled levels of mechanical loading. Some evidence in chondrocytes suggests that physiological mechanical signals stimulate production and secretion of growth factors, substance P, and IL-4 that mediate extracellular matrix synthesis and remodelling [3]. CNP additionally mediates matrix response through regulation of cGMP regulated ion channels (CGi) [51]. These cyclic nucleotide-gated channels belong to the superfamily of voltage-gated ion channels that regulate membrane potentials and could evoke calcium (Ca^2+^) entry in chondrocytes [53,54]. We previously showed that the stretch-activated (SA) ion channels and the integrins mediate mechanical loading induced calcium signaling and regulate anabolic and catabolic pathways in chondrocytes [3,28,55,56]. The contrasting effects are due in part to different temporal dynamics and magnitude of the kinases and transcription factors, which are influenced by the cytokines or mechanical loading. Thus, further studies should examine the molecular pathways induced by CNP and mechanical signals in inflammatory chondrocytes. The proposed interactions of CNP with mechanical signals are illustrated in Figure 5.

In summary, CNP treatment leads to significant increases in anabolic activities and the reduction of catabolic mediators in the presence of IL-1β. The anabolic response was PKGII mediated and could be enhanced by mechanical signals in a synergistic manner. The catabolic response was not influenced by the PKGII inhibitor, implicating alternative mechanisms involving the PDEs. Further studies will examine the relation between CNP and mechanical loading in detail, because these signals converge on a common mediator, cGMP. For instance, because NO is an established player in mediating both mechanical and inflammatory signals in cartilage, we will determine which components of the CNP and mechanical loading pathways interfere with the cytokine-induced NO pathway and whether these factors directly regulate each other or cross-talk with other signaling routes.

## Conclusions

Therapeutic agents like CNP could be administered in conjunction with controlled exercise therapy to slow OA disease progression and maintain cartilage health. The findings from this research provide the potential for developing a novel agent to slow the pathophysiologic mechanisms and to treat OA in the young and old.

## Abbreviations

cGMP: 3,5'-cyclic guanosine monophosphate; CNP: C-type natriuretic peptide; GC-B: guanylyl cyclase B; IL-1β: interleukin-1β; NO: nitric oxide; PDE: cGMP-regulated phosphodiesterase; PGE_2_: prostaglandin E_2_; PKGII: cyclic GMP-dependent protein kinase II.

## Competing interests

The authors declare that they have no competing interests.

## Authors' contributions

MR and TC carried out the experiments and analysis, participated in the experimental design, data analysis, and manuscript drafting. DL and DS participated in the experimental design, data analysis, and manuscript drafting. All authors read and approved the final manuscript.
